# Daunorubicin induced Stevens‐Johnson syndrome: A case report

**DOI:** 10.1002/ccr3.4475

**Published:** 2021-07-16

**Authors:** Preeti Shakya, Amit Sharma Nepal

**Affiliations:** ^1^ B.P. Koirala Memorial Cancer Hospital Bharatpur Nepal; ^2^ Institute of Medicine Tribhuvan University Kathmandu Nepal

**Keywords:** chemotherapy, daunorubicin, hypersensitivity reaction, Stevens‐Johnson syndrome/toxic epidermal necrolysis

## Abstract

Clinicians should consider the possible association of Daunorubicin with Stevens‐Johnson syndrome (SJS), administer it with caution and promptly evaluate all subsequently developing cutaneous reactions with a high index of suspicion for Stevens‐Johnson syndrome.

## Graphical abstract

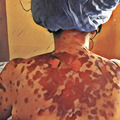

## 1 INTRODUCTION

According to the extent of widespread epidermal detachment, Stevens‐Johnson syndrome/toxic epidermal necrolysis (SJS/TEN) is classified as SJS, SJS/TEN overlap, and TEN. SJS is the less severe condition, in which skin detachment is less than 10 percent of the body surface area (BSA). TEN involves detachment of more than 30 percent of BSA. SJS/TEN overlap describes patients with skin detachment of 10‐30 percent of BSA. The majority of cases stemming from SJS/TEN are the result of hypersensitivity reactions. These are due to drugs such as anticonvulsants, sulfonamide antibiotics, antiretrovirals, non‐steroidal anti‐inflammatory drugs (NSAIDs), allopurinol, and corticosteroids.^1^, ^2^, ^3^, ^4^ The potential association of anticancer agents with SJS/TEN has not been systematically investigated and has been inconsistently reported. Daunorubicin is an anthracycline antibiotic that has antineoplastic activity and is used in the therapy of acute leukemia and AIDS‐related Kaposi sarcoma, with side effects that are reportedly negligible^5^. In this report, we describe a fatal case of a 2‐year‐old boy with precursor B‐cell acute lymphoblastic leukemia/lymphoma (ALL) who developed SJS due to Daunorubicin.

## 2 CASE REPORT

A 2‐year‐old male child diagnosed with precursor B‐cell ALL underwent a multi‐drug regimen remission induction chemotherapy as per the modified Berlin‐Frankfurt‐Münster (BFM) 90 ALL protocol. This consisted of vincristine 1.4mg/m2/dose intravenously once a week (D1,8), prednisolone 40mg/m2/day orally daily (D1‐8), L‐asparaginase 6000 Units/m^2^/dose intravenously q.a.d from D2 (a total of 3 doses), Methotrexate 8 mg/dose intrathecally once a week (D1,8) and Daunorubicin 30 mg/m^2^/dose intravenously on D8.

About 2 weeks after the initiation of the first induction chemotherapy, the patient developed cutaneous erythema around the face extending to the chest with ulceration of mucosal surfaces of the oropharynx which quickly progressed into confluent erythematous and necrotic eruption with blistering of the skin.

**FIGURE 1:**
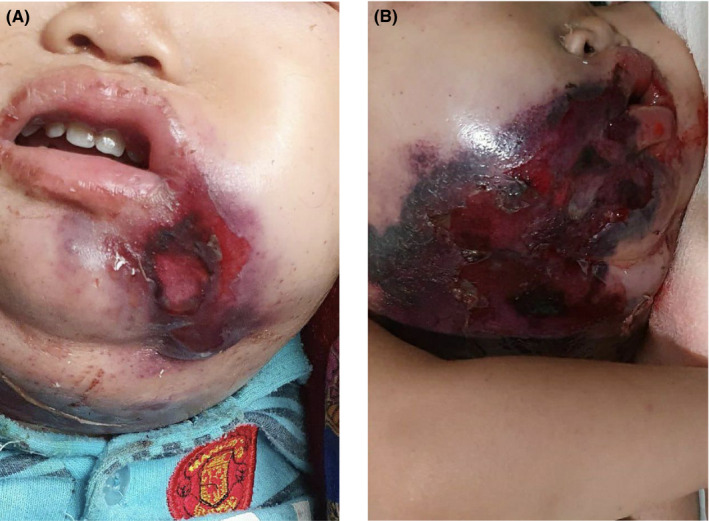
Skin changes in Stevens‐Johnson syndrome: (A) Initial erythematous rash with sloughing of mucosa over the angle of mouth. (B) Necrotic purpuric rash with sloughing of the skin with mucocutaneous involvement of lips, face, and neck

On systemic examination, the patient was febrile and there was the presence of vesiculobullous lesions over the face and neck region, sloughing of the lips and oral mucosa, and within the oral cavity covering less than 10% body surface area (Figure 1A and B). Nikolsky's sign was positive.

The diagnosis of SJS was made on clinical grounds by a dermatological consultation.

His complete blood count suggested pancytopenia (hemoglobin—10 g/dl, total leukocyte count‐700/Ul, platelet count‐8000/Ul). His blood investigation suggested severe neutropenia (Absolute Neutrophil Count −35). His serum albumin level was noted to be 2.8 g/dl and his serum alkaline phosphatase was 306 U/L. His urine routine examination showed 3+ urine sugar. Other biochemical test results were within normal limits initially.

The patient was admitted to the intensive care unit. A blood culture was sent which was positive for *Pseudomonas aeruginosa* and the patient was started on supportive antibiotics with injection Vancomycin and Gentamicin. In view of decreased counts, injection filgrastim 50 μg was given subcutaneously once a day. The other supportive cares included wound care, fluid and electrolyte management, nutritional support, ocular care, temperature control, and pain management.

After stopping the chemotherapy, the patient's rash started to improve. The patient developed hospital‐acquired pneumonia a few days after which was managed accordingly. Despite the intervention, the patient developed sepsis and he succumbed to his illness on the 6th day. The cause of death was reported to be septicemia.

## 3 DISCUSSION

The mechanism underlying SJS remains unknown in patients administered with combination chemotherapy. In our case, we believed that the patient's problem was related to one of the four chemotherapy drugs since the syndrome was not present before the initiation of chemotherapy, suggesting that it was not part of the presenting features of his disease.

Although SJS occurs in a very small percentage of patients who use chemotherapy drugs, several reports have demonstrated a potential association of anticancer drugs with SJS and TEN, including conventional cytotoxic and novel targeted agents.^6^ In our case, the last administered drug to the patient was Daunorubicin which according to FAERS (Food and Drug Administration Adverse Effect Reporting System) has very few reported cases of SJS.^6^ We believe Daunorubicin to be the culprit for SJS in our patient as the immediate withdrawal of Daunorubicin led to the improvement in signs and symptoms.

Daunorubicin forms the cornerstone of treatment in the remission induction of both adult and childhood acute leukemia. A liposomal formulation of Daunorubicin is available as first‐line therapy for AIDS‐related Kaposi sarcoma. It is a cytotoxic antibiotic that acts by intercalating between DNA base pairs and uncoiling the DNA helix, which results in inhibition of DNA synthesis and apoptosis of rapidly dividing cells. Common side effects of Daunorubicin include myelosuppression, nausea, vomiting, mucositis, diarrhea, alopecia, red urine, and cardiotoxicity. Cutaneous side effects are very rare and include reversible alopecia, rash, contact dermatitis, and urticaria.^7^ The possible association of Daunorubicin with SJS has not been well‐established and inconsistently reported.

The main difficulty in attempting to identify an offending agent in chemotherapy is the frequent co‐administration of multiple anticancer agents. Furthermore, the administration of drugs that are known to have strong associations with these cutaneous reactions—(ie sulfonamide antibiotics, corticosteroids, and allopurinol) adds to the difficulty.^1^, ^2^, ^3^, ^4^ In the majority of published case reports, patients were exposed to multiple agents.

In our case, the patient was also co‐administered corticosteroids as part of the remission induction chemotherapy. Although, there are few reports suggesting corticosteroids as an offending agent of SJS/TEN, their role as triggers is not clearly defined and there is a high potential for confounding by several factors, including the use of anti‐infective and anticonvulsant agents, a history of radiotherapy, and a history of collagen vascular disease.^3^, ^8^

The mucocutaneous complications of anti‐cancer drugs are widely observed in oncology and hematology departments, but clinicians/oncologists may overlook these reactions because of the severity of the underlying primary disorder itself. In conclusion, it is very critical but complicated to identify the offending agents when oncologic patients develop SJS during chemotherapy. It is crucial to be able to differentiate life‐threatening cutaneous adverse reactions that require immediate management from more benign manifestations of chemotherapy and treat the same as an oncologic emergency. The potential association of Daunorubicin with SJS should be considered and administered with caution. As soon as the first suspicious sign of SJS/TEN is observed, the offending drug must be discontinued immediately, aggressive medical management must be started or the patient must be referred to appropriate centers promptly.

Early recognition and intervention can significantly alter the course of the disease and improve the outcome. Identification and prompt removal of precipitating factors are the most pivotal steps for the lifesaving management of SJS.

## 4 CONCLUSION

Clinicians should consider the potential association of Daunorubicin with SJS, administer it with caution and observe patients closely for potentially dangerous cutaneous reactions and treat it as an oncologic emergency. If multi‐drug regimen chemotherapy is employed, one must administer the drugs with caution and all subsequently developing cutaneous reactions must promptly be evaluated with a high index of suspicion for SJS.

## AUTHOR CONTRIBUTIONS

Preeti Shakya: Conceptualization (lead); Writing—Original draft (lead), Writing—review and editing (equal). Amit Sharma Nepal: Critical revision of the article (lead), Writing—review, and editing (equal). Both authors contributed to the final version of the manuscript.

## ETHICAL APPROVAL

The ethical issues were completely considered to prepare this case report according to our institution's ethical board guidelines. Moreover, this article was prepared regarding the Declaration of Helsinki.

## CONSENT STATEMENT

Published with written consent of the patient.

## Data Availability

Data sharing is not applicable to this article as no new data were created or analyzed in this study.
